# Aging and Hearing with Vestibular Schwannoma Beyond Presbycusis: A Large Cross‐Sectional Audiometric and Volumetric Study

**DOI:** 10.1002/oto2.70272

**Published:** 2026-06-29

**Authors:** Sami Barrit, Mejdeddine Al Barajraji, Salim El Hadwe, Jérome R. Lechien, Daniele Morelli, Cécile Renier, Nicolas Massager

**Affiliations:** ^1^ Department of Neurological Surgery CHU Tivoli La Louvière Belgium; ^2^ Institut de Neurosciences des Systèmes Aix‐Marseille Université Marseille France; ^3^ Research Department Science New York USA; ^4^ Department of Neurological Surgery University of California San Francisco USA; ^5^ Computational Precision Health University of California Berkeley California USA; ^6^ Faculty of Medicine UMONS Research Institute for Health Sciences and Technology, University of Mons (UMONS) Mons Belgium; ^7^ Department of Neurosurgery CHR Citadelle Liege Belgium; ^8^ Department of Clinical Neurosciences University of Cambridge Cambridge UK; ^9^ Department of Surgery UMONS Research Institute for Health Sciences and Technology, University of Mons (UMons) Mons Belgium; ^10^ Department of Otolaryngology and Head and Neck Surgery, Foch Hospital, School of Medicine, UFR Simone Veil Université Versailles Saint‐Quentin‐en‐Yvelines (Paris Saclay University) Paris France; ^11^ Department of Otolaryngology and Head and Neck Surgery CHU Saint‐Pierre Brussels Belgium; ^12^ Department of Otolaryngology Elsan Hospital of Poitiers Poitiers France; ^13^ Department of Radiophysics CHU UCL Namur‐Site Hôpital Sainte‐Elisabeth Namur Belgium; ^14^ Faculty of Medicine Université Libre de Bruxelles Brussels Belgium

**Keywords:** acoustic neuroma, hearing preservation, internal auditory canal, natural history, vestibular schwannoma

## Abstract

**Objective:**

To identify predictors of auditory function in treatment‐naïve vestibular schwannomas (VS) while accounting for age‐related hearing loss, investigating baseline volumetrics and audiometrics in a pre‐intervention stereotactic radiosurgery (SRS) cohort.

**Study Design:**

Cross‐sectional study.

**Setting:**

Single‐center national SRS referral program with a prospective database.

**Methods:**

All consecutive patients with newly diagnosed unilateral VS and complete pre‐intervention imaging and audiometric data were included. High‐definition MRI and bone CT with co‐registration and fusion enabled morphometric and volumetric analysis of VS and internal auditory canal (IAC) dimensions. Univariate tests, multivariate logistic regression with interaction terms, and regularized regression identified factors associated with serviceable hearing (Gardner‐Robertson scores 1‐2).

**Results:**

Among 533 patients (50.65% male, mean age 57.04 years), 72.6% had serviceable hearing. Median VS volume was 875.3 mm^3^ (IQR: 328.6‐1732.5). The final multivariate model (AUC = 0.719) retained age (OR = 0.920, *P* = .0144), canal filling ratio (CFR; OR = 0.465, *P* = .0446), and a significant age × intracanalicular‐to‐total tumor volume ratio interaction (*P* = .0318). Age‐stratified analyses revealed that the CFR–hearing relationship reversed across age groups: in younger patients (<40 years), higher canal filling was associated with better hearing preservation (94% vs 73%), while this pattern reversed in older patients (>60 years). Younger patients also demonstrated greater canal dilation (*P* = .002), though this lacked independent predictive value (*P* = .366).

**Conclusion:**

While age dominates hearing prediction in VS, canal filling ratio significantly modifies this relationship in an age‐dependent manner, representing a clinically accessible imaging biomarker with age‐specific thresholds and effects.

Vestibular schwannomas (VS), or acoustic neuromas, originate from Schwann cells of the vestibular portion of the eighth cranial nerve. Despite their benign histology, they cause significant morbidity through compression of adjacent neural structures within the internal auditory canal (IAC) and cerebellopontine angle. Progressive sensorineural hearing loss, the most common presenting symptom, results primarily from cochlear nerve involvement.^1^ Understanding factors influencing auditory function is therefore essential for prognostication, patient counseling, and treatment planning.

Management encompasses observation with serial imaging and audiometry, microsurgical resection, and stereotactic radiosurgery (SRS).^2^ Advances in neuroimaging have increased VS detection rates, yielding diagnoses of smaller tumors at earlier stages in patients with minimal symptoms,^3^ making the distinction between observation and early intervention increasingly important.

Previous research has identified several factors potentially associated with VS‐related hearing loss, including age and presbycusis,^4^ tumor characteristics (size, volume, growth rate), and anatomical relationships with the IAC.^5^ Tumor growth rate may predict hearing decline during observation more powerfully than initial size,^6^ yet hearing deterioration can occur without documented tumor growth.^7^ High‐resolution neuroimaging now enables precise examination of the tumor–IAC relationship,^8^ with attention to the proportion of tumor within the canal, canal filling,^9^ and bony changes such as IAC widening.^10^ Canal filling has been proposed as a quantitative measure of tumor occupancy, potentially reflecting nerve compression at the fundus. Tumors extending to the lateral canal end may impact hearing more than those preserving a cerebrospinal fluid buffer (the “fundal cap”). Additionally, VS secretions—particularly inflammatory cytokines—can biochemically injure the inner ear and cochlear nerve, potentially explaining hearing decline with minimal nerve compression.^11^, ^12^ The natural history of VS‐related hearing loss thus likely involves a complex interplay of mechanical, anatomical, and biological mechanisms.^11^, ^13^

Despite these advances, studies distinguishing clinically meaningful predictors from incidental associations remain scarce, limited by cohort sizes and population heterogeneity. Here, we leverage a uniquely large, homogeneous cohort of newly diagnosed, treatment‐naïve VS patients from an SRS program, who typically present with less advanced disease than surgical candidates, providing insights into the natural history at earlier stages representative of the growing subpopulation with extensive management options requiring accurate prognostication.

This study addresses two key questions: (i) How do specific VS characteristics, including volumetric distribution and IAC parameters, influence hearing when properly accounting for age‐related hearing loss?; and (ii) Can we identify clinically relevant thresholds providing evidence‐based guidance for management decisions?

## Methods

All patients from a single‐center SRS program's prospective database with newly diagnosed unilateral VS and complete standardized imaging and audiometric studies before any intervention were included. Patients with neurofibromatosis or bilateral VS were excluded. The Ethical Committee of ULB approved the study (ref. P2015/207), and all patients provided informed consent.

Each patient underwent same‐day 1‐mm‐thick axial three‐dimensional MRI (gadolinium‐enhanced T1‐weighted and T2‐weighted sequences without intersectional gaps) and 1‐mm‐thick axial three‐dimensional CT. Imaging was integrated in Leksell GammaPlan™ (Elekta Instruments®) for co‐registration and volumetric study.

All VS were graded according to Koos classification (I‐IV). Grade I tumors are purely intracanalicular; grade II extend into the cerebellopontine angle without brainstem contact; grade III contact the brainstem without compression; grade IVa cause brainstem displacement without fourth ventricle distortion; grade IVb cause significant displacement with fourth ventricle distortion.

We measured ipsilateral (IIAC) and contralateral (CIAC) internal acoustic canal maximal length and height, then performed slice‐by‐slice manual contouring of both IAC and VS to compute volumes. A single investigator segmented intra‐ and extra‐IAC volumes, measuring total lesion volume (tLV), intracanalicular lesion volume (icLV), and extracanalicular lesion volume (ecLV). Derived parameters included the intracanalicular‐to‐total tumor volume ratio (icLV/tLV), canal filling ratio (CFR; icLV/IIACV), and canal dilation ratio (CDR; IIACV/CIACV). Auditory function was assessed using pure‐tone audiometry and speech discrimination testing, classified by the Gardner‐Robertson (GR) scale, with serviceable hearing defined as GR scores of 1 or 2.

### Statistical Analysis

#### Primary Approach

Normality was assessed using the Shapiro‐Wilk test. Mann‐Whitney *U* tests compared continuous variables between hearing groups (all nonnormally distributed); categorical variables were analyzed with Chi‐square or Fisher's exact tests. Correlation analysis used Spearman's rank coefficient. Multivariate analysis assessed multicollinearity using variance inflation factors (VIF > 10 excluded). A baseline logistic regression with age alone was followed by sequential models incorporating significant morphometric variables. Interaction terms were tested via likelihood ratio tests. The final model was evaluated using ROC/AUC analysis, with classification performance assessed at the optimal threshold from Youden's *J* statistic.

#### Secondary Approaches

LASSO regression with 5‐fold cross‐validation and Elastic Net regularization addressed feature selection challenges from multicollinearity. Restricted cubic splines explored nonlinear relationships, with models compared using AIC. Threshold analysis on key morphometric variables used sequential cutpoints evaluated with Youden's *J* statistic, supplemented by inflection point analysis. Age‐stratified threshold analyses divided patients into 3 groups (<40, 40‐60, >60 years) to identify age‐specific thresholds. Odds ratios with 95% confidence intervals were calculated for age‐specific logistic regression models, along with comprehensive clinical utility metrics including sensitivity, specificity, predictive values, risk difference, and number needed to treat (NNT).

#### Advanced Modeling

Machine learning algorithms (Random Forest, Gradient Boosting, Support Vector Machines) were validated using 10‐fold stratified cross‐validation, with feature importance assessed through permutation importance and SHAP values. IAC remodeling was analyzed by categorizing CDR into quartile‐based groups with stratified models. Mediation analysis tested whether IAC dilation mediates the tumor volume–hearing relationship using bootstrap confidence intervals. Age confounding was addressed through age‐stratified analyses, non‐linear age modeling (quadratic terms and splines), and propensity score matching. Sensitivity analyses assessed robustness to outlier removal, alternative model specifications, and different inclusion criteria. Visual clinical decision support tools integrating age, CFR, and tumor distribution were developed for risk stratification.

All analyses used Python 3.8 with scikit‐learn, statsmodels, scipy, matplotlib, and seaborn.

## Results

The study included 533 patients with untreated VS, of whom 72.6% had serviceable hearing. Median age was 56 years (range 18‐89), with 50.65% male. Tumor laterality was evenly distributed (50.09% right‐sided). Koos grade distribution: grade I 25.1% (134/533), grade II 32.3% (172/533), grade III 36.6% (195/533), grade IVa 6.0% (32/533). Median tLV was 875.3 mm^3^ (IQR: 328.6‐1732.5). Details are provided in Table 1.

**Table 1:** Univariate Analysis of Clinical, Tumor, and Canal Parameters Associated with Hearing Status

Variable		Overall (n = 533)	Serviceable (n = 387)	Unserviceable (n = 146)	*P*‐value	Test
Age (years)		57.04 ± 13.30	54.89 ± 12.99	62.73 ± 12.46	0	*t*‐test
IIACV (mm^3^)		288.71 ± 149.77	284.81 ± 132.54	299.05 ± 188.13	.328	*t*‐test
IIAC length (mm)		8.22 ± 2.02	8.14 ± 1.85	8.43 ± 2.42	.135	*t*‐test
**IIAC height (mm)**		**6.34** ± **2.04**	**6.20** ± **1.93**	**6.71** ± **2.28**	.009*	***t*‐test**
**tLV (mm^3^)**		**1246.24** ± **1576.44**	**1115.72** ± **1465.68**	**1592.20** ± **1798.13**	.002*	***t*‐test**
icLV (mm^3^)		173.98 ± 139.22	167.14 ± 121.36	192.09 ± 177.33	.065	*t*‐test
**ecLV (mm^3^)**		**1072.26** ± **1541.85**	**948.58** ± **1431.84**	**1400.11** ± **1765.07**	.002*	***t*‐test**
CIACV (mm^3^)		198.73 ± 66.48	200.20 ± 67.05	194.85 ± 65.03	.407	*t*‐test
CIAC length (mm)		6.94 ± 1.52	6.93 ± 1.47	6.97 ± 1.64	.806	*t*‐test
CIAC height (mm)		4.84 ± 1.18	4.81 ± 1.18	4.89 ± 1.19	.491	*t*‐test
CDR		1.48 ± 0.63	1.46 ± 0.57	1.54 ± 0.76	.153	*t*‐test
**icLV/tLV**		**0.41** ± **0.33**	**0.43** ± **0.33**	**0.36** ± **0.33**	.038*	***t*‐test**
IAC height ratio (ipsi/contra)		1.36 ± 0.47	1.34 ± 0.46	1.41 ± 0.50	.112	*t*‐test
**CFR**		**0.57** ± **0.24**	**0.56** ± **0.23**	**0.61** ± **0.25**	.029*	***t*‐test**
Sex ratio (male:female)		270:263	196:191	74:72	1	Chi‐square
Laterality (right:left)		266:267	200:187	66:80	.216	Chi‐square
**Koos grade**	**I**	**134 (25%)**	**102 (26%)**	**32 (22%)**	.001*	**Chi‐square**
	**II**	**172 (32%)**	**135 (35%)**	**37 (25%)**	.001*	**Chi‐square**
	**III**	**195 (37%)**	**135 (35%)**	**60 (41%)**	.001*	**Chi‐square**
	**IVa**	**32 (6%)**	**15 (4%)**	**17 (12%)**	.001*	**Chi‐square**

Values are presented as mean ± standard deviation for continuous variables and n (%) for categorical variables. Serviceable hearing was defined as Gardner‐Robertson (GR) class 1‐2; unserviceable hearing as GR 3‐5. Group comparisons were made with independent‐samples *t*‐tests for continuous variables and *χ* ^2^ tests for categorical variables; the corresponding test is indicated in the “Test” column. Koos grade reflects tumor size and extension. Bold values indicates statistically significant.

Abbreviations: IAC, internal auditory canal; mm, millimeters; mm^3^, cubic millimeters; V, volume.

*P < .05 denotes statistical significance.

**Figure 1:**
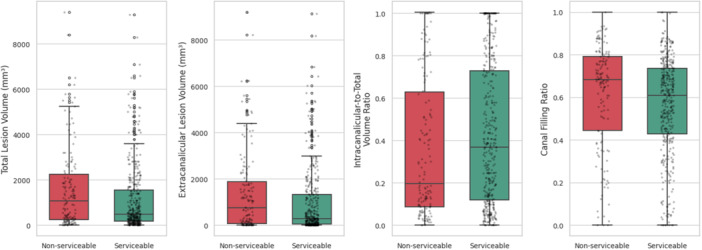
Distribution of key predictors by hearing status. Boxplots comparing total lesion volume, extracanalicular lesion volume, intracanalicular‐to‐total volume ratio, and canal filling ratio between nonserviceable (red) and serviceable (green) hearing groups. *P < .05, **P < .01, ***P < .001.

Univariate analysis identified age as a strong predictor (*P* < .0001; rho = 0.3899 with GR score). The base age‐only model yielded an odds ratio of 0.951 (95% CI: 0.936‐0.967), indicating a 4.9% decrease in odds of serviceable hearing per additional year. Significant tumor/canal predictors included tLV (*P* = .0042), ecLV (*P* = .0057), intracanalicular‐to‐total volume ratio (*P* = .0214), and CFR (*P* = .0035) (Figure 1). Koos grade was significantly associated with hearing.

**Figure 2:**
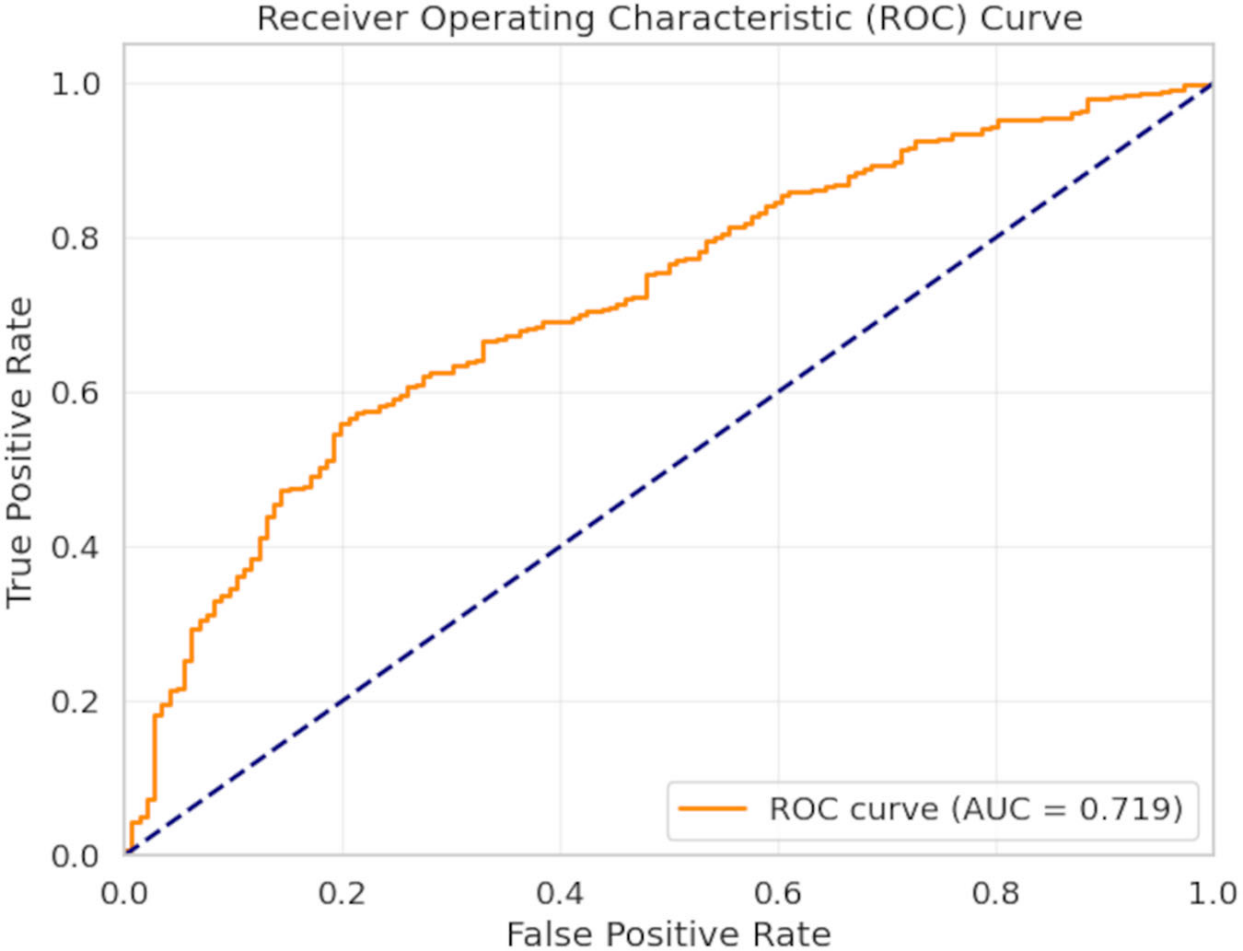
ROC curve for the final multivariate model predicting serviceable hearing (AUC = 0.719). At the optimal threshold of 0.760, sensitivity was 0.561 and specificity was 0.801.

Multivariate analysis revealed substantial multicollinearity, necessitating careful variable selection. The final model (AUC = 0.719 vs 0.683 for age‐only; Figure 2) incorporated a significant age × intracanalicular‐to‐total volume ratio interaction (*P* = .0318; *χ* ^2 ^= 4.87, *P* = .0273). This interaction indicates that a higher intracanalicular proportion is associated with maintained hearing in younger patients, while this relationship attenuates or reverses in older patients. Significant predictors: age (OR = 0.920, 95% CI: 0.861‐0.983, *P* = .0144), intracanalicular‐to‐total ratio (OR = 0.076, 95% CI: 0.006‐0.967, *P* = .0476), CFR (OR = 0.465, 95% CI: 0.220‐0.984, *P* = .0446), and the age × ratio interaction (OR = 1.032, 95% CI: 1.003‐1.062, *P* = .0318) (Table 2).

**Table 2:** Multivariate Logistic Regression Models for Prediction of Serviceable Hearing

Variable	Base Model	Extended Model	Final Model (with interaction)
Age	0.951 (0.936‐0.967), ***P*** < .0001*	0.953 (0.937‐0.969), ***P*** < .0001*	0.920 (0.861‐0.983), ***P*** = .0144*
Intracanalicular‐to‐total tumor volume ratio	‐	1.824 (0.923‐3.602), ***P*** = .0838*	0.076 (0.006‐0.967), ***P*** = .0476*
Canal filling ratio	‐	0.473 (0.227‐0.985), ***P*** = .0455*	0.465 (0.220‐0.984), ***P*** = .0446*
Age × Intracanalicular‐to‐total tumor volume ratio	‐	‐	1.032 (1.003‐1.062), ***P*** = .0318*
AUC	0.683	0.701	0.719
Sensitivity	0.742	0.711	0.561
Specificity	0.521	0.596	0.801

Values are presented as odds ratio (95% confidence interval), *P*‐value. Bold values indicates statistically significant.

Abbreviation: AUC, area under the receiver operating characteristic curve.

*P < .05 denote statistical significance.

**Figure 3:**
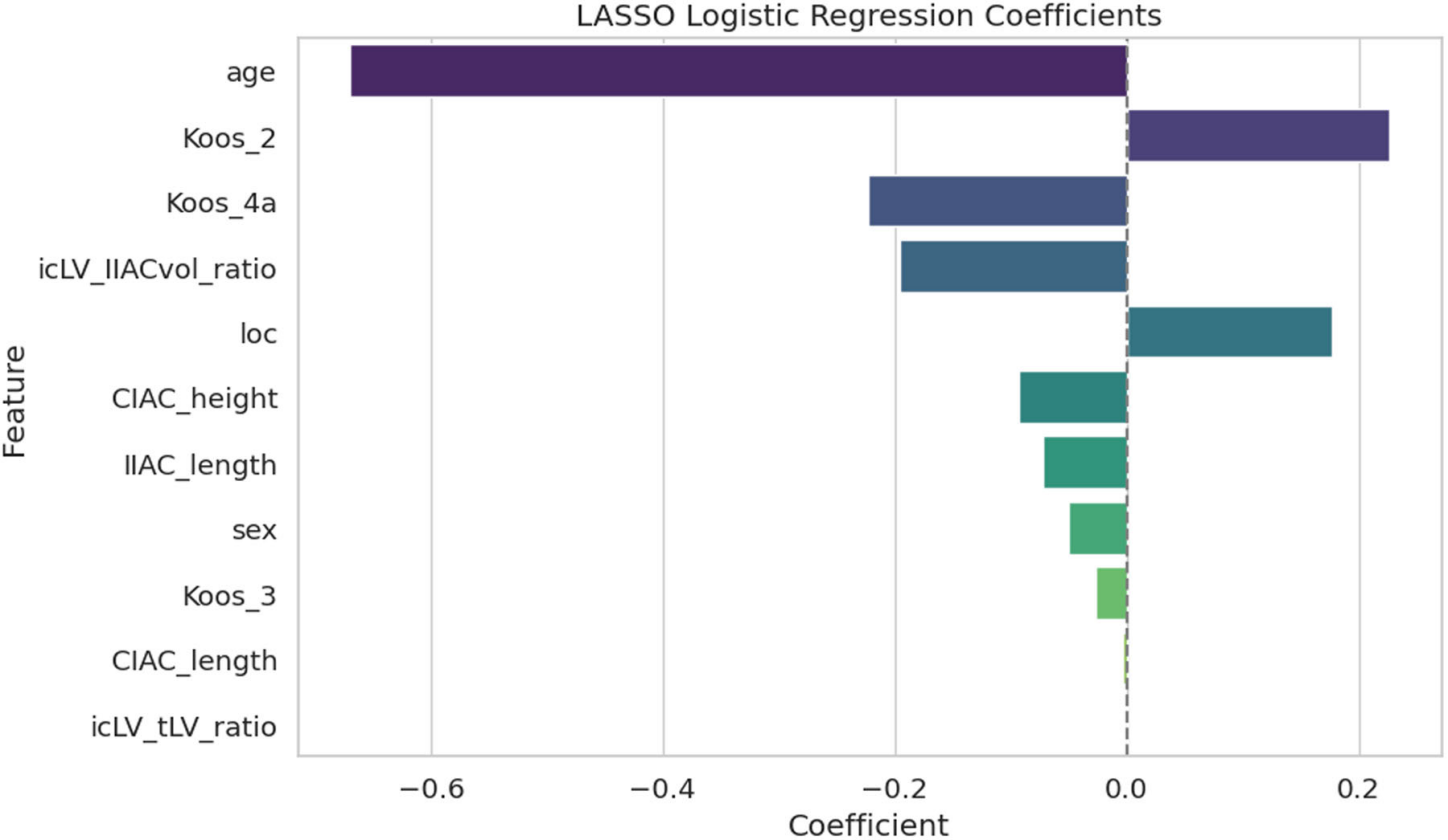
LASSO regression coefficients predicting serviceable hearing. Age shows the strongest negative association; Koos grade 2 shows a positive association. Canal filling ratio (icLV/IIACV) demonstrates a substantial negative coefficient.

LASSO regression confirmed age as the strongest predictor (coefficient = −0.670), followed by Koos grade 2 (0.227), Koos grade 4a (−0.224), and CFR (−0.196) (Figure 3). Machine learning approaches corroborated these findings.

**Figure 4:**
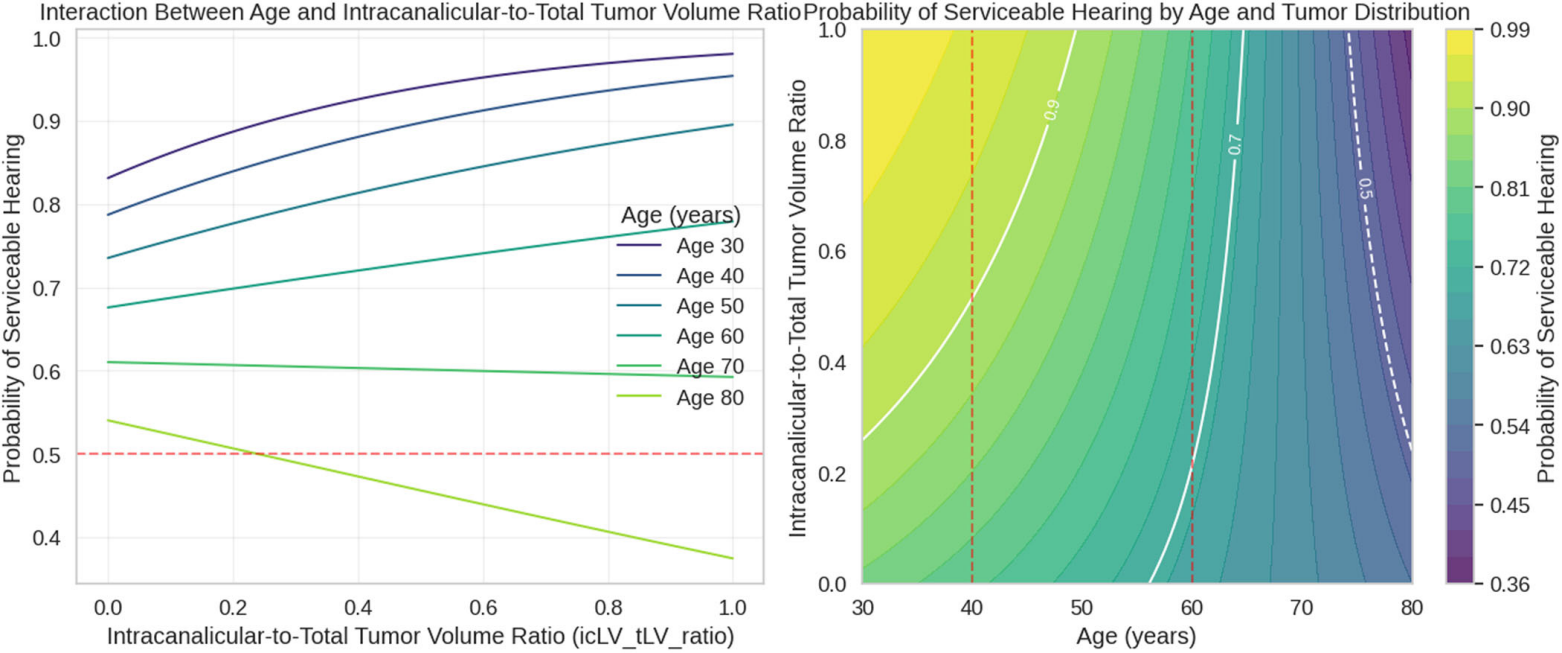
Age‐tumor distribution interaction effects on serviceable hearing probability. Left: age‐specific curves for intracanalicular‐to‐total volume ratio. Right: probability heatmap with contour lines (0.5, 0.7, 0.9) across age and tumor distribution.

**Figure 5:**
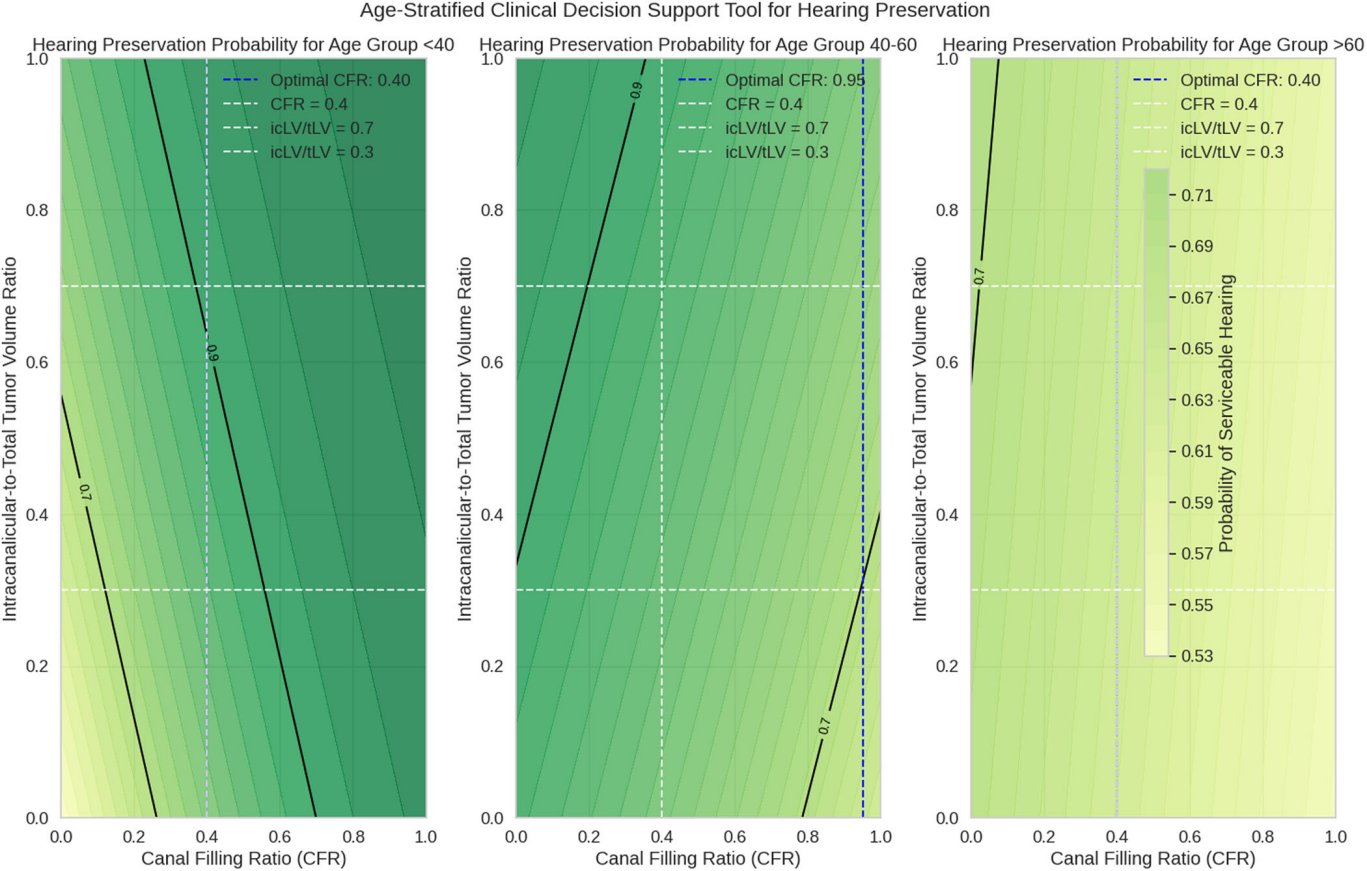
Age‐stratified clinical decision support tool. Probability maps of serviceable hearing by canal filling ratio and intracanalicular‐to‐total volume ratio for age groups <40, 40 to 60, and >60 years. Blue dashed lines indicate optimal CFR thresholds.

Threshold analysis identified an overall optimal CFR cutpoint of 0.40. Age‐stratified analyses revealed the strongest discriminatory power in young patients (<40 years: threshold 0.40, Youden's *J* = 0.448) versus middle‐aged (40‐60: threshold 0.30, *J* = −0.007) and older patients (>60: threshold 0.40, *J* = 0.039) (Table 3). The CFR–hearing relationship fundamentally reversed across age groups: in younger patients, higher CFR was associated with better preservation (moderate filling 94.4%, high filling 94.1% vs low 73.3%), while in older patients, moderate filling showed better outcomes (71.0%) than high (53.2%) or low filling (55.8%). For middle‐aged patients, tumor distribution pattern was particularly significant (*P* = .0114), with mixed patterns yielding the best outcomes (89.6%) (Figures 4 and 5).

**Table 3:** Canal Filling Ratio Age‐Stratified Threshold Analysis

Age group	Optimal threshold	Sensitivity	Specificity	Youden's *J*
All patients	0.40	0.458	0.562	0.020
<40 years	0.40	0.750	0.698	0.448
40‐60 years	0.30	0.333	0.674	−0.007
>60 years	0.40	0.444	0.595	0.039

Youden's *J* = Sensitivity + Specificity − 1; higher values indicate better discriminatory ability.

CDR analysis revealed age‐dependent patterns: younger patients showed greater canal dilation (severe dilation mean age 53.5 vs minimal 58.6 years, *P* = .002), though association with hearing outcomes was not significant (*P* = .366).

## Discussion

This large cross‐sectional study of 533 treatment‐naïve VS patients confirms age as the dominant predictor of baseline auditory function while demonstrating that tumor characteristics—particularly intracanalicular‐to‐total volume ratio and CFR—significantly modify this influence. Our multivariate model showed statistically significant improvement over an age‐only model; however, the absolute AUC increase remained limited and should be interpreted cautiously. This incremental gain does not imply immediate clinical applicability as a standalone decision tool. Rather, these results are best viewed as descriptive and hypothesis‐generating, highlighting imaging features contributing to baseline hearing phenotypes within a radiosurgical context. Age‐sensitive analyses revealed different optimal CFR thresholds across age groups, with strongest discriminatory power in younger patients, suggesting fundamentally different pathobiological processes potentially requiring tailored approaches.

These findings nuance current understanding. Previous studies have inconsistently linked hearing outcomes to tumor–host parameters. Matthies et al^14^ reported associations between IAC diameter differences and preoperative deafness without controlling for confounders. Tsunoda et al^15^ found no IAC‐cochlear damage correlation, while Badie et al^16^ and Lapsiwala et al^17^ established connections between intrameatal pressure and hearing impairment in small samples (15‐40 patients). Brown et al^18^ found tumor size and location failed to consistently correlate with hearing loss, while Berrettini et al^19^ suggested intracanalicular tumors caused earlier loss. We previously demonstrated that icLV strongly predicts IAC dilation.^8^ This inconsistency is predominantly explained by limited statistical power, heterogeneous populations, and varied selection biases. Neglected age‐dependent relationships may have also played a significant role; the intracanalicular‐to‐total ratio demonstrated a statistically significant age interaction, but its modest odds ratio represents a clinically subtle effect easily missed in smaller cohorts.

The reversal of CFR effects across age groups, coupled with greater canal dilation in younger patients, points toward age‐dependent tumor‐host interactions. Younger patients with higher CFR maintained better hearing while exhibiting greater dilation, but this reversed in older patients. This may be explained by a physiological compensation threshold model: adaptive mechanisms in younger patients effectively preserve function up to a limit, beyond which compensatory capacities become insufficient, suggesting a non‐linear relationship differing from the more gradual deterioration in older patients. Greater CDR in younger patients—a structural change not expected to reverse—suggests several possibilities: more aggressive endophenotypes enhancing bone remodeling, intrinsically higher biological remodeling capacity, or earlier tumor development allowing more extensive remodeling. These observations must be interpreted cautiously given our cross‐sectional design and potential selection biases. CFR shows stronger discriminatory power in younger patients but limited utility in older ones where presbycusis predominates; CDR lacked independent predictive value after multivariate adjustment.

For middle‐aged patients, our analyses revealed a distinct transitional pattern between younger compensatory mechanisms and older presbycusis‐dominated profiles. Tumor distribution pattern emerged as particularly significant, with mixed distribution associated with better outcomes, suggesting spatial distribution of tumor burden may become more influential than simple volumetric parameters during middle age. The age‐dependent findings may also reflect mechanisms beyond mechanical compression; tumor secretions and inflammatory cytokines may biochemically injure the cochlea with age‐varying effects. In older patients, the non‐linear CFR–hearing relationship (moderate filling showing better outcomes than either low or high filling) suggests complex interactions between aging auditory pathways and tumor effects.

A similarly designed study of a surgical cohort^20^ provides informative comparison. Their cohort was younger (mean 48 vs 57 years) with substantially larger tumors (4260 vs 1246.24 mm^3^) at more advanced grades (Koos III‐IV: 72.8% vs our I‐III: 94%), yet showed remarkably similar serviceable hearing rates (74.8% vs 72.6%). While Dörner et al^20^ found tumor volume independently predictive in multivariate analysis, we identified intracanalicular‐to‐total ratio as more important through its age interaction. Their linear IAC widening measurements showed uniform negative hearing associations, while our volumetric CDR analysis revealed age‐dependent patterns without significant hearing associations. Our advanced statistical techniques—interaction terms and regularized regression—captured subtleties that traditional models cannot detect. These complementary findings suggest canal parameters influence hearing with greater complexity than previously recognized, particularly in patients with early‐stage disease and extended management options.

Current guidelines acknowledge age generally^2^, ^21^, ^22^, ^23^ but lack age‐sensitive insights for interpreting imaging biomarkers. Our findings suggest identical tumor characteristics carry different prognostic implications depending on age beyond presbycusis alone. For clinicians, this translates to a straightforward principle: high CFR in a young patient should not raise the same concern for hearing deterioration as in an older patient. While current evidence is insufficient for immediate guideline changes, future research could lead to nuanced recommendations recognizing age as both a treatment fitness consideration and a marker of diverse pathobiological mechanisms.

Several limitations warrant consideration. This SRS‐eligible cohort does not encompass the full VS management spectrum, potentially under‐representing very small or very large tumors, and may be influenced by center‐specific referral patterns. SRS eligibility was not determined by fixed thresholds, though these factors naturally informed treatment decisions. Findings should be interpreted as characterizing baseline audiometric–volumetric associations within an SRS‐treated population at radiosurgical planning. The cross‐sectional design precludes causal inference and assessment of pre‐treatment disease duration. Some statistically significant associations have uncertain clinical relevance given modest effect sizes. Manual contouring may introduce observer variability, though all segmentations were performed by one experienced neurosurgeon using a standardized approach. Fundal cap involvement was not assessed. Gardner‐Robertson classification may not capture subtle audiometric patterns. The limited number of younger patients may reduce stability of age‐stratified estimates. Concurrent hearing conditions and molecular/genetic factors were not investigated.

Future research would benefit from longitudinal studies tracking canal parameters and hearing evolution in broader treatment‐naïve cohorts, establishing causality and determining whether age‐dependent patterns represent true pathobiological differences or selection artifacts, while validating age‐specific thresholds in independent cohorts. Methodological refinements—incorporating granular audiometric data and fundal cap assessment—would strengthen investigations. Multicentric collaboration is necessary, requiring standardized protocols for imaging, segmentation (including formal intra‐rater reliability assessment), and outcomes reporting to facilitate pooled analyses.^24^, ^25^, ^26^ Integration of molecular biomarkers with longitudinal audiometric and volumetric data may help distinguish mechanical from biochemical contributions across age groups, ultimately enabling identification of actionable endotypes and implementation of validated, phenotype‐specific clinical decision support tools.

## Conclusion

This study demonstrates that while age remains the dominant hearing predictor in VS, tumor characteristics—particularly CFR—modify this effect with age‐dependent clinical significance in a large SRS‐selected cohort. Through high‐precision volumetric and age‐sensitive analyses, we report CFR as an imaging biomarker whose interpretation substantially differs across age groups, strongest in younger patients and progressively weaker with advancing age. The fundamental reversal of CFR effects suggests distinct pathobiological processes requiring different clinical approaches. Future management guidelines may benefit from age‐specific interpretation of radiographic parameters, integrated within comprehensive VS management considering facial nerve preservation, tumor control, vestibular function, and therapeutic benefit–risk assessment. Research harmonization and multicentric longitudinal studies are needed to validate these patterns and establish age‐stratified clinical thresholds with therapeutic implications.

## Author Contributions

**Sami Barrit**, conceptualization, methodology, formal analysis, data curation, visualization, writing—original draft, writing—review and editing; **Mejdeddine Al Barajraji**, visualization; **Salim El Hadwe**, methodology, formal analysis, data curation, investigation, writing—review and editing; **Jérome R. Lechien**, supervision, methodology, writing—review and editing; **Daniele Morelli**, investigation, data curation, writing—review and editing; **Cécile Renier**, methodology, formal analysis, statistical analysis, writing—review and editing; **Nicolas Massager**, conceptualization, supervision, project administration, writing—review and editing.

## Disclosures

Previous Presentations: Data of this study has not been previously presented nor published.

### Competing interests

None.

### Funding source

None.
